# Co-therapy in Open Dialogue: Transforming therapists’ self in a shared space

**DOI:** 10.3389/fpsyg.2023.1083502

**Published:** 2023-01-25

**Authors:** Christina Lagogianni, Eugenie Georgaca, Dimitra Christoforidou

**Affiliations:** ^1^School of Psychology, Aristotle University of Thessaloniki, Thessaloniki, Greece; ^2^School of Humanities and Social Sciences, University of Thessaly, Volos, Greece

**Keywords:** Open Dialogue, co-therapy, professional self, experiencing self, self-transformation, dialogic practice

## Abstract

The present study aimed to explore co-therapists’ relationship and how therapists’ individual presence influences this relationship in Open Dialogue. Although co-therapy is key in Open Dialogue network meetings, the processes of that relationship remain largely understudied. The study applied thematic analysis to semi-structured interviews with 20 Open Dialogue trained therapists working in public and private sectors internationally. The results indicate that therapists are present in a meeting with their experiencing and professional self. Specific co-therapy processes allow co-therapists to attune to one another verbally and physically, creating a shared space that promotes new common understandings, shared responsibility and ultimately a transformation of each therapist’s self and practice. Trust between co-therapists seems to be a prerequisite for co-therapy to flourish. Results of the present study reveal a dynamic influence of co-therapy practice, in which co-therapy promotes a more dialogical personality and allows the therapists’ own transformation, which in turn enables common understandings and sharing of responsibility. Considering the growing interest in dialogical approaches and Open Dialogue trainings, trainers, supervisors, and practitioners need to be aware of and attend to the dynamics of co-therapy relationship in order to care for themselves, their team and ultimately the networks they collaborate with.

## Introduction

Open Dialogue is a philosophical and therapeutic approach of being with people in times of crisis/need, as well as a way of organizing mental health services based on network meetings (Olson et al., 2014; Putman, 2022b). Network meetings involve a team of at least two professionals, the person of concern and his/her social network, namely relatives, friends, colleagues or other service members already engaged in the individual’s care (Putman, 2022b). In Open Dialogue meetings practitioners of different professional backgrounds come together to form inter-agency groups as a way to promote polyphony (Seikkula et al., 2001; Olson and Seikkula, 2003). Professionals’ teams often include, among others, psychiatrists, psychologists, occupational therapists, psychiatric nurses, social workers and experts by experience, known as peer workers (Nelson et al., 2022; Razzaque et al., 2022). For the purposes of the present study, all professionals involved in network meetings will be referred to as “therapists.” Different professional backgrounds, with diverse ways of meaning making, may influence therapists’ reflections with their co-therapist and their dialogue with the network (Holmesland et al., 2014).

Co-therapy, meaning two or more therapists working together in sessions with clients or families, has been developed in the field of family therapy in clinical and supervision settings and is in the heart of Open Dialogue practice (Ast et al., 2019). Although it is acknowledged that the quality of co-therapists’ relationship influences treatment (Borchers et al., 2013), the prospects and challenges involved in co-therapists’ relationship remain largely understudied in the field of Open Dialogue literature. As every relationship is unique, when collaborating with different co-therapists, practitioners might share different aspects of themselves and thereby allow diverse opportunities for the network members to explore. In appreciating the importance of each therapist presence and use of self in therapy, it is worth exploring how co-therapists experience their relationship, and how they attend to their self while promoting continuity of care with the networks. The present study aims to contribute to the limited dialogue around co-therapists’ relationships and the processes involved, expanding, thus, on the literature of co-therapy in the Marriage and Family therapy field and the Open Dialogue approach.

Co-therapy has been widely practiced in Marriage and Family therapy, enriching the professional role of the therapists, as it can offer more resources, alternative perspectives and hypotheses (Hannum, 1980; Hendrix et al., 2001; Reed, 2013). Through the ways co-therapists collaborate and talk to each other, they can act as a role model for couples and families for alternative ways of communication patterns (Hannum, 1980; Hendrix et al., 2001). Co-therapists complement each other through alternating roles, from active to reflective positions, support each other to maintain a neutral stance and avoid being absorbed by the family dynamics, qualities that were believed to be helpful in systemic practice (Selvini et al., 1980; Benjamin and Benjamin, 1994).

In ensuring these qualities, training and supervision practices have been recognized as key to allow the space for therapists’ professional development following the co-therapy relationship (Hendrix et al., 2001). A non-competitive and united team is likely to contribute to the growth of both clients and therapists (Selvini et al., 1980). To achieve this, it is important that each therapist acts as a host to both the clients and their co-therapist, making each therapist a host and guest at the same time. Considering therapists as both hosts and guests in a meeting is in line with suggestions that having two therapists allows for the clients’ greater sense of continuity and permanence, while also preventing therapists’ burnout (Hoffman et al., 1995). It also points to the importance of exploring the relationship between therapists, as well as the supervision and/or in session practices that allow this hosting experience to be cultivated.

Even though co-therapy has been recognized as an effective and often constructive practice in the Marriage and Family therapy literature, it has also received some criticism. Besides practical challenges in terms of time demands and increased cost, challenges regarding use of co-therapy might arise when co-therapists have control issues with each other, an erotic relationship, and when clients are trapped in co-therapists’ symbolic therapeutic parenting (Russell, 1980; Bowers and Gauron, 1981; Haley, 1987; Hendrix et al., 2001). These concerns are highly valuable and point to the importance of co-therapists being both self-aware but also attentive and caring of the relationship with each other, to assist the network of concern. Trainees collaborating with different co-therapist dyads commented that they found challenging the possibility that, when working with a co-therapist, there is an increased likelihood to learn something new about themselves (Hendrix et al., 2001). Although the authors did not further discuss this, it is possible that, when collaborating with co-therapists with different levels of experience, issues around control and hierarchy might arise, increasing levels of complexity and the possibility of unexpected issues emerging in a meeting. From a dialogic point of view this might be perceived as an opportunity rather than a challenge, as therapists’ self-attentiveness and being aware of their emotional reactions in therapy can offer valuable insights to the network and the process of therapy (Rober, 1999).

In dialogical practices, co-therapy builds on therapists’ non expert position and focuses on more relational characteristics in a network meeting, as a means to encourage dialogue (Seikkula et al., 2012; Hornova, 2020). Dialogue is understood as a joint process that develops within network meetings through promoting a language that opens new flows of questions and new discourses (Seikkula, 1995). Co-therapy is inspired by and inspires in turn the seven core principles of Open Dialogue, both in how services are organized – assisting in immediate help, inviting the social network, having flexibility and mobility, maintaining responsibility and psychological continuity – and in the way of being with people – tolerating uncertainty and dialogism (Olson and Seikkula, 2003). “Two or more therapists in a team meeting” is the first of the twelve fidelity elements to dialogic practice (Olson et al., 2014). Having multiple therapists in a team meeting with a network supports the development of polyphony, through promoting alternatives and giving space to different voices (Valtanen, 2019; Hornova, 2020). Open Dialogue practitioners perceive dialogical co-therapy as a process that entails unique relational qualities, including the ability to disagree with each other, willingness to be challenged in therapy, taking care of the co-therapists’ relational space, and finally being aware of and talking about embodied responses (Hornova, 2020).

During Open Dialogue meetings, therapists tend to respond to networks’ experiences on an embodied and verbal level (Shotter, 2011; Cromby, 2012; Borchers et al., 2013; Kykyri et al., 2017; Seikkula et al., 2018). When therapists share their feelings, using their affective responses and their embodied experiences, their co-therapist is likely to do the same and ‘contaminate’ this way of talking to the whole network (Borchers et al., 2013; Hornova, 2020). In this way, dialogical co-therapy allows greater body-awareness and self-reflexivity (Hornova, 2020). Growing research in the ‘Relational Mind in Events of Change in Multi-actors Therapeutic Dialogues’ reveals an embodied synchrony in the physiological responses of members of the network and the therapists (Karvonen et al., 2016; Päivinen et al., 2016; Kykyri et al., 2017; Seikkula et al., 2018; Laitila et al., 2019). Interestingly the co-therapists appear to have the highest level of synchrony with each other, highlighting the importance of attunement between co-therapy dyads (Karvonen et al., 2016). Despite the recognition of embodied attunement and the influence of co-therapy on a personal level (Borchers et al., 2013), the processes through which co-therapists manage to tune in to each other and influence each other’s presence remain largely understudied.

Using a dialogical loop of co-therapists’ interviews and a focus group to increase credibility of the emerging themes around co-therapy, Hornova (2020) revealed that dialogical co-therapy is perceived as energizing for therapists. This might be related to the ability of dialogical practitioners to be themselves in meetings with families, which further creates a feeling of satisfaction (Sidis et al., 2020). Still, to be authentic in voicing the therapist’s inner dialogue and emotions can be difficult for health care workers, as this might require an expansion of the professional role (Holmesland et al., 2014). Open Dialogue meetings often challenge practitioners, by demanding a role release and role expansion of their original professional training, i.e., as psychiatrists, psychiatric nurses, social workers etc. (Holmesland et al., 2014). Such mental health trainings typically encourage developing professionals to be in charge of their emotions and keep them to themselves (Rowan and Jacobs, 2011). Therapists discern meaning based on filters, constructed in different schools of thought and professional trainings. Those filters may block or magnify resonances of therapists with their clients and influence the way they respond to them.

Reflecting on the role of psychiatrists in multi-professional teams, Valtanen (2019) acknowledged the importance of trust and shared understanding among team members. Within a feeling of shared understanding, co-therapists can disagree with their partner. Instead of perceiving it as competition, disagreeing with one’s co-therapist is viewed as a way to develop polyphony of equal voices and a way to be authentic (Hornova, 2020). In a similar context, that of Need Adapted Treatment of psychosis with two or more co-therapists present, psychiatrists being interviewed through co-research practices (Andersen, 1997) and stimulated recall interviews (Kagan et al., 1963) recognized that in a treatment situation they are present not only as professionals but also as individuals who share an individual relationship with their co-therapist (Borchers et al., 2013). It has also been found that, when having a personal relationship with one’s co-therapist and knowing the personal difficulties they are encountering, therapists are more inclined to perceive their co-therapists as patients themselves and take care of them (Borchers et al., 2013). Although this promotes a safe and friendly working environment, it might present challenges to the roles and responsibilities therapists take on. Creating open spaces for discussions between therapists may help bridge their differences, produce a shared professional identity and cultivate the feeling of safety in the co-therapists’ relationship (Holmesland et al., 2014; Valtanen, 2019; Hornova, 2020).

Therapists in an Open Dialogue meeting are not only “hosts” or “guests” of the session but part of the unique encounter of the session, willing to be equally transformed through the therapeutic relationship (Olson and Seikkula, 2003; Brown et al., 2015; Kykyri et al., 2017; Hornova, 2020). This is one of the reasons Open Dialogue trainings include supervision and family of origin groups in their core as a way to appreciate the theoretical underpinnings of Open Dialogue through practice and personal involvement (Putman, 2022a). To be willing to be transformed in a meeting requires self-attentiveness and responsiveness, properties that are cultivated in turn through supervision practices and therapy (i.e., family of origin). It is argued that these requirements of dialogical trainings change and shape significantly practitioners’ perceptions of their self and their professional role in therapy (Von Peter, 2019, 2021; Pocobello, 2021; Hendry et al., 2022). Although co-therapy might come up as a theme in supervision and training contexts, not enough attention is given to the relationship of co-therapists, the ways that co-therapists collaborate and manage their differences, and how this willingness to transform might be present and experienced by co-therapists and the network.

In line with Open Dialogue, the present paper follows Bakhtin’s (1984) view of the self as polyphonic, comprising of different voices. Developing research reveals the rich inner conversation of therapists during sessions, including attending to client process, processing the client’s story, focusing on therapist’s own experiences and managing the therapeutic process (Rober et al., 2008). Therapists are invited to attend to all voices in the room, appreciate the horizontal polyphony between network members and the professional team, as well as the vertical polyphony within themselves and each individual in turn (Seikkula, 2008). Co-therapists have an active role in constructing the therapeutic reality as members of the given context and facilitators of the therapeutic process. Following this, co-therapists are not perceived as the experts and their ideas are not imposed to the clients, but they may act as stimuli for change (Andersen, 1991; Rober, 1999, 2005b; Anderson, 2005). As members of the therapeutic encounter, therapists co-create the safe space for the network to unfold their narratives and find words for the not yet said (Shotter, 2011, 2015). In this relational space, therapists’ own experience is crucial and may act as a compass to navigate around the multiple voices in a network meeting. Therapists’ lived experience involves their use of self, their positioning, body changes, emotional reactions, thoughts, values and beliefs (Simon, 2012; Miller and Baldwin 2013; Avdi and Seikkula, 2019; Gkantona, 2019; Ong et al., 2021; Aponte, 2022). The ways therapists use their experiences is associated with being mindful of their internal process and aware of the influences these may have on the therapeutic process (Rowan and Jacobs, 2011; Mojta et al., 2014).

In order to appreciate the polyphony of their inner voices, therapists need to be attuned to and reflect on both their professional self and their experiencing self (Rober, 2005b; Borcsa and Janusz, 2021). The “professional self,” influenced by therapist’s skills, training and professional development, takes an observer position and is conceptualized as the inner voice of the therapist that hypothesizes and responds to clients’ stories (Rober, 2005b). The “experiencing self*,”* a more intimate self, refers to memories, images and fantasies associated to these observations, drawn from therapists’ personal experiences (Rober, 2005b). As the experiencing self is related to therapists’ feelings and personal story being evoked during a meeting, it is argued that therapists’ own therapy is key in their attunement and use of the experiencing self (Simon, 2006; Clark, 2009; Flaskas, 2009). Through the process of one’s own therapy and/or personal growth practices a greater self-awareness and attentiveness is developed (Lum, 2002; Miller and Baldwin 2013).

Therapists’ professional self and experiencing self are in an ongoing inner conversation during a meeting, providing different opportunities to respond to their co-therapist’s and the networks’ invitations and stories (Seikkula et al., 2012; Borcsa and Janusz, 2021). Through alternating between facilitating and reflective positions in the external dialogue with the network and engaging in the reflective processes co-therapists allow the space to each other to attend to their inner dialogue, become more attuned to their inner voices and ultimately be able to develop polyphony (Seikkula, 1995; Rober, 1999, 2005b; Olson and Seikkula, 2003). Differences in therapists’ reflexivity and attention to their professional and experiencing selves may influence not only their own presence in a meeting but also co-therapy practice and ultimately provision of care with the networks (Georgaca, 2012; Αvdi and Georgaca, 2018). Exploring how the differences between therapists’ professional and experiencing selves influence co-therapy can enhance our understanding of co-therapy practice and Open Dialogue network meetings.

Since training in Open Dialogue and dialogic presence have become increasingly popular, it is important that therapists acknowledge those opportunities and appreciate the complexity of the relationship with their co-therapists. The present study aims to contribute to the understanding the co-therapists’ relationship and how this relationship influences the individual presence of each therapist. For the purposes of the research the concepts of professional self and experiencing self will be used, to capture part of therapists’ inner dialogue and vertical polyphony. It is assumed that if more light is shed into how co-therapists interact with each other while attending not only to their own presence but also to their co-therapist presence as a way to connect and be with the network, more constructive and supportive practices will be developed.

The research questions are: (a) *How is therapists’ professional self and experiencing self present during co-therapy?* and (b) *What are the co-therapy processes that influence therapists’ self?*

## Materials and methods

### Design

The present study aims to examine what aspects of the therapist’s self are mobilized during co-therapy, how the therapist’s self is affected by co-therapy and which co-therapy processes influence the therapist’s self. The study is part of a wider project concerning Open Dialogue practitioners’ views and experiences of co-therapy. Two consecutive interviews were conducted with Open Dialogue practitioners, using distinct semi-structured interview guides. The present study was facilitated by the lead author (CL) and the second study, that is still in process, focused on dialogical practices of co-therapists, and was facilitated by the third author (DC). Although the research was done in collaboration, the two studies were analyzed and written separately.

The first author (CL) was completing the 3 years training in Open Dialogue UK at the time and that allowed her access to related practitioner networks. The conceptualization and the interview schedule used in the present study was influenced by the first author’s experience of working with different co-therapists and by reflections in the supervision context of the training. The third author (DC) has collaborated with the Mental Health team of Volos, Greece, the first public service in Greece that has been using Open Dialogue informed practices since 2009. The team dynamics and the development of the approach in that context inspired the third author to explore further co-therapists’ relationship. Upon completion of each interview the two researchers reflected on their experience and provided feedback to each other for the interviews to follow. The two authors have been collaborating as co-researchers in this process, allowing space for reflexivity and ongoing reflection in the development of the interview guides, approaching participants, implementation of the interviews, and analysis.

### Participants

Purposive sampling was used by contacting Open Dialogue international institutes and advertising the research in the closed Facebook group ‘Network for Open Dialogue and Reflective Processes’. The selection criteria were that participants had to have completed training in Open Dialogue and have experience working with co-therapists.

Participants were 20 Open Dialogue therapists, eight male and 12 female. According to the professional identities that participants introduced themselves with, eight were psychologists, two psychiatrists, three social workers, one nurse, one peer worker and five therapists did not mention a specific mental health background. It is worth mentioning that often participants’ professional roles involved more than the above titles; additional roles included being trainers in Open Dialogue and having administrative positions. Participants worked both in the public sector and in private practice. They came from various geographical locations; 10 participants came from the European Union, five from the United Kingdom, three from the United States of America and two from Australia. Participants’ Open Dialogue experience ranged from three to 20 years. They had practiced co-therapy with two to thirty different colleagues.

### Data collection

Participants’ views and experiences of co-therapy were generated and recorded through semi-structured individual interviews lasting 45–60 min using the online conference platform Google Meet. Before the interview participants completed a demographics form and signed a consent form, confirming knowledge of the confidential and anonymous nature of the data, their right to withdraw and their acceptance to record the interview. In the beginning of the interview the two researchers (CL, DC) introduced each other as co-researchers and allowed time for questions. One researcher would interview and the other was taking a reflective position. Researchers recognized that the one taking the reflective position, waiting for her turn to conduct her research, could engage in the interview if needed. This, however, did not happen at any point of the data collection process. The interview schedule for this study started with questions regarding the ways in which the participants’ professional background, namely professional roles and previous training, influence the reflections in a meeting. Participants were also asked what it means to be authentic in a network meeting and how their own personal therapy, personal growth practices, family of origin and other therapies might have influenced that. Finally, there were questions regarding the ways co-therapists support each other to be authentic and respond fully as embodied persons in a network meeting. Participants were encouraged to provide clinical examples for their experiences.

### Analysis

All interviews were transcribed, meticulously read, and annotated for important themes and common patterns emerging across interviews by the first author (CL). Thematic analysis was used, aiming to identify themes that capture important aspects of the research question (Braun and Clarke, 2006). All data were coded without trying to test a specific hypothesis, but rather to depict contributors’ experiences, applying an inductive thematic analysis. A line-by-line coding was conducted through reading and annotating the first two interviews. Preliminary lists of codes were created, in which codes captured participants’ thoughts, experiences, feelings and images on the matter of investigation. Interviews three and four added to the list of codes and created new codes, when contributors’ perspectives were new. The same process was followed for interviews 5 to 20. To ensure that the interviews coded last were attended equally to the first interviews, the codes developed were revised many times, reflecting an ongoing back and forth involvement with the data set and coding process. In searching for themes, the codes were listed and grouped based on their commonalities. A name and description were given by the first author to these themes.

Reviewing the themes and adjusting the names and descriptions of themes was accomplished by extensive validation sessions with the second (EG) and third author (DC), who also acted as inter-coders of selected extracts, to promote accuracy and transparency in the coding process. No inter-agreement measures were used in this process; instead, consensual validation and agreement procedures were followed. In each validation session the first author presented the themes and justified them with reference to participants’ quotes. Then through collaborative discussions on differing views, authors reached consensus on the themes best capturing participants’ experiences.

The first (CL) and third author (DC) both interacted with all participants of the present study during interviewing, something which allowed them experiential insight into the data collected. They are also both practicing clinicians, and this allowed them a more practice-relevant perspective on the data. The second author (EG) is an academic, experienced in research and initially less attached to the data set. The different perspectives on the same data by the three researchers allowed a variety of voices to emerge when evaluating authors’ positions and expectations of the study, while ensuring richness and reflexivity in the process of generating themes (Tong et al., 2007; O’Connor and Joffe, 2020).

As a result of the analytical process, the data were organized into three main themes, namely *therapist’s individual presence*, *co-therapy processes*, and *co-therapy as a shared space*, each consisting of different subthemes. In addition, a fourth theme emerged, *trust as a prerequisite*, that connects all three themes.

## Results

The themes and subthemes that emerged will be presented and described below, accompanied by representative extracts from the interviews.

### Therapist’s individual presence

Therapists are present in a network meeting, bringing both their experiencing and professional selves. Having completed a dialogical training, the therapist’s presence of experiencing and professional self is already changed following supervision and personal therapy training requirements.

#### Presence of experiencing self

1. **Use of embodied responses**: The vast majority of participants recognized that part of bringing their experiencing self in a meeting involves being aware of and sharing their own embodied experiences and responses.
  ‘My understanding of embodied experience is that it invites us to stay in touch with our mind. […] I can first make a little note to myself that, okay this builds a tension in me and even maybe, digging a bit deeper in that, noticing it, but not maybe feeling that it is a good idea to share it in words, but just noticing and breathing deeper and knowing that it affects my co-worker too, how am I in this moment, how am I holding it in a way’ (P8).
2. **Stronger when therapist is self-aware**: It was commonly acknowledged that family of origin, personal therapy and self-growth practices of therapists allow them to be more self-aware and notice their experiencing self in a meeting. Some participants recognized that through their own personal growth journey therapists are more aware of their feelings, vulnerabilities, blind spots, and traumas. A participant commented that this helps to be more humble and curious, not rushing to judge others, as they become aware that all families, including their own, can have ‘*breakdowns in their communication’* (P15). A different voice emerged from a participant who noted that the mandate of therapists being self-aware may mean that they *‘use the power that they are given working in psychiatry and mental health, while exploring their own humanity and experiences on that’* (P10).
3. **Focus remains on the network**: Some participants recognized the importance of self-reflexivity when sharing their experiencing self, talking about their feelings and ideas gently in a way that the network can say no. When they consider that self-disclosure may be helpful for the network, therapists may even explicitly share their personal stories and resonances, and ‘*connect them to the network’s narrative’* (P1). When thinking through the connection between the network’s needs and the therapist’s experiencing self, one participant noted that co-therapy might not always be the preferred practice for all network members; one to one therapy might be preferable, as a way to explore more private issues and build individuals’ confidence.
4. **Involves greater ownership**: Some participants shared the view that through attending to and reflecting on their experiencing self, a sense of ownership is developed that helps therapists ‘*trust the feeling that is being evoked*’ (P14). A participant who is a psychologist stressed the therapist’s responsibility toward themselves: ‘[One has to be] *responsible for their own emotions and ideas in a meeting, and to give voice to them without competing over their co-therapist’s*’ (P17).

#### Presence of professional self

1. **Invite professional role**: All participants agreed that the school of thought or training of therapists does not define therapists in an Open Dialogue meeting. They all added that different kinds of professional training are perceived as competences of practitioners and are taken into account when the team is formed, as a way to best adapt to the network’s needs. Participants working in multi-professional teams, mainly in the public sector, acknowledged that they often decide on their co-therapists based on their professional background and/or invite other co-workers to consult their network meetings considering their professional background and the needs of the network.
2. **Expertise as part of the polyphony**: Almost all participants recognized that, although part of their contribution to the dialogue may come from their professional role, their expertise, therapists tend to pull back from the expert position and offer all their ideas in a more tentative way in acknowledging that all voices in a meeting are important. More than half of participants used the same wording to characterize working with different therapists of diverse professional backgrounds as allowing for ‘*horizontal polyphony*’, ‘*richness in understanding*’ and ‘*more opportunities in a meeting*’ (P1, P4, P6, P9, P11, P12, P14, P16, P17, P19, P20). Ideas from the therapist’s professional self become another voice in the meeting rather than the prominent way of exploring the network’s story. Therapists can be more attuned to different parts of an individual’s narrative, depending on their professional background, i.e.,: policies around risk.
  ‘Part of what I enjoy about doing this work, that I can say - Ah, that’s a different approach, I wouldn’t have thought about it that way. But sometimes I may say in a reflection – That’s really interesting, I wonder what the family members think, we should ask them. - It’s almost if I cannot work it out between us, then I will use the family as a resource’ (P12).
3. **Stronger when there is uncertainty**: A different voice, expressed by some participants, argued that the presence of many voices may create uncertainty in a meeting. When therapists feel uncertain, they might fall back to a more directive approach, to techniques and understandings deriving from their expertise. They may, thus, return to their professional identity, that is familiar and feels safer to sit with, and ‘*hear what we have learnt’* (P12).

### Co-therapy processes

Participants pointed out specific processes that they engage in with their co-therapists that allow them to tune in to each other and to the network.

#### Balance through reflection

Most participants acknowledged that the presence of a co-therapist during times of crisis and uncertainty can help tolerate the polyphony and allows thinking about emotions in a more reflective way. Some argued that this allows them to balance their positions and emotions with their co-therapist, by slowing down, sharing their concern and searching for differences rather than sameness in their reflections.

‘I think having a co-therapist can be very helpful if people become polarized in their position. And I can easily become polarized in my position, so having a co-therapist who can sometimes say - I feel you are stuck where you are, I am not sure what is happening for you - and so being able to challenge each other’ (P14).

#### Invitational language

Like in discussions with the network, some participants saw the language between co-therapists as needing to be invitational, careful, and gentle. This invitational language was considered important for different reasons for each contributor. A few participants argued that when their co-therapist invites them to share the reasons they self-disclosed a personal story or a feeling helps them unfold their thinking, make direct connections to the network and keep the dialogue around the network rather than themselves. One participant said that invitational language allows promotion of trust: ‘*I have to trust that you are not going to share it inappropriately. And they have to trust that you will not push them beyond their limits’* (P7).

#### Knowing and accepting each other

Almost all participants shared the view that when co-therapists get to know each other a gentler, more careful and caring attitude toward one’s co-therapist is created. In order to be authentic and express their thoughts and feelings it is important that the therapists feel ‘*sufficiently safe and comfortable’* with their co-therapist (P20). For many participants it was important that they felt unconditionally accepted by their co-therapist, meaning that there were no expectations, no right or wrong ways to be in a meeting. Two participants mentioned that being comfortable refers not only to ways of expression but also to the style of the co-therapist partner, for instance staying in silence or the use of humor. Therapeutic style is distinguished from professional training, as it refers to the way therapists are with people and foster containment. Some participants argued that it might be likely that they cannot work with specific co-therapists, even if they know them well, as co-therapy is a human relationship that, like all relationships, might not work well in particular cases.

#### Attuning verbally and physically

Getting to know one’s co-therapist allows noticing them changing in a non-verbal way during a meeting. Almost half of the participants saw the embodied responses in a meeting as a channel to connect with their co-therapist. A couple of participants mentioned that noticing these embodied reactions in themselves or their co-therapist can be a useful starting point for reflections between co-therapists or an appreciation that someone else has changed in the session, creating more space for the not yet said. A participant said that they sometimes practice mindfulness with their co-therapist before starting a network meeting as a way to “*be attuned to each other” (P7).*

#### Taking care of the relationship

Dialogic relational spaces between co-therapists outside network meetings were viewed as important by all participants, as they allow deepening their level of attunement with each other. Co-therapists need further spaces to talk about themselves and how it is for them working together. Participants gave different examples of how such dialogical spaces can be created, including before meetings, in supervision or in post session reflections. Almost all participants considered supervision spaces as core in allowing for discussions regarding the relationship of co-therapists. Two participants further acknowledged the need for supervision as a way to discuss how it feels to be challenged, an experience that is quite rare in everyday interactions. Most participants saw supervision practices as a way to avoid competition between co-therapists, which was acknowledged as a threat to co-therapy practice.

‘We have to make a decision when we are in a network meeting: can we discuss this here, or has something been triggered in us that’s too negative, that we maybe have to take to our supervision, because it is something about our relationship, not the family, that we might need to take somewhere else to manage’ (P14).

### Co-therapy as a shared space

Through those co-therapy processes a shared space between co-therapists is constructed that promotes novel common understandings, a sense of shared responsibility and ultimately a transformation of each therapist’s self and practice.

#### Common understandings based on different perspectives

Spaces to talk about co-therapists’ relationship and experience of working together promote a shared attitude toward clients and the network. More than half of participants mentioned that when working with their co-therapists they create common understandings by co-constructing narratives based on each other’s experiences, while respecting their differences. A few participants consider those common understandings as the premise for an open conversation with the network, as they promote safety in therapeutic encounters. For some, common understandings contributed to a feeling of shared hope that things can change for the better. Below is an example of an open dialogue trained therapist collaborating with a drama therapist:

‘She [drama therapist] was very focused on creating the drama, I suppose, you know the scene. And I would actually introduce a different way of thinking about it. She was setting up a scene for this young man who was quite unwell, to be able to connect with his unusual belief. And the mother was there, as well, and I was able to draw in, What do you think your mother thinks about this or What do you think your father might say about this? So I created a more open dialogue about it, where she was sort of focused on creating the experience for the client’ (P16).

#### Shared responsibility

Many participants recognized that the ability of different professionals to become more flexible and open in a meeting depends on the power and responsibility attributed to them in their training. Some participants viewed psychiatrists and psychoanalysts as being traditionally trained to have greater responsibility and a sense of certainty and knowing during a session. For these professionals, stepping away from the expert position can be quite a step away from their professional training. Most participants agreed that sharing responsibility and believing that co-therapists are in the process of supporting the network together allows the development of open relationships and dialogue with the network. A few participants mentioned that a collaborative non-hierarchical relationship is being formed, that is not based on the therapists’ original training.

#### Transformation of therapists’ self and practice

The vast majority of participants saw co-therapy processes and the relationship with the co-therapist as transforming the therapist’s way of perceiving their practice as well as their experiencing and professional self. Therapists’ professional identity seems to be radically reexamined in rethinking the expert position and recognizing the professional role as one of the many voices in therapists’ inner polyphony. In addition, there was overall agreement between participants that co-therapy processes help in opening space for their experiencing self to unfold, as they are being invited to share their lived experience in a meeting rather than just their professional judgment. Not only the way that therapists practice therapy changes, but also the ways they attend to their own inner polyphony is enriched, through their co-therapist’s invitations, the reflective processes and observing their co-therapist. Some participants noted that collaborating with a co-therapist who attends to their own embodied experience helps the therapist to do the same, thereby being more attentive to their own experiencing self. ‘*You are not just listening to words, you are taking everything that is happening*’ (P16).

### Trust as prerequisite

The importance of trust was recognized for all participants as a prerequisite for the co-therapists’ relationship to emerge and unfold. Still, different participants approached it in diverse ways. Some claimed that co-therapists need to hold the space for each other, in a similar way they hold the space for the network. Some participants saw trust in one’s co-therapist including knowing that they will respond in a gentle way, while having good intentions. Others considered that trusting one’s co-therapist allows their experiencing self to emerge and to be vulnerable in sessions. Some participants conceptualized trust and respect for one’s co-therapists as a way to allow tolerating being openly challenged on a professional and personal level. For a few participants having been trained with their co-therapists and having a shared experience in the family of origin group allowed to build a trusting relationship. For almost all participants, co-therapy is perceived as a process that can cultivate trust. In this sense, trust is not fixed in stone but rather is constantly constructed between co-therapists. Greater trust in the relationship between co-therapists allows for increased trust in the dialogical process and in the network.

## Discussion

The present study aimed to explore the influence of therapists’ professional and experiencing self on co-therapy. It also sought to examine the processes involved in co-therapy and how these shape therapists’ selves. As co-therapy is one of the key elements of Open Dialogue meetings, the way therapists’ self is implicated in a meeting is likely to impact the collaboration between co-therapists and subsequently the ability to adapt to the needs of the network (Borchers et al., 2013). Participants’ testimonies are in line with previous research on co-therapy in the context of Open Dialogue (Borchers et al., 2013; Holmesland et al., 2014; Hornova, 2020) and with theoretical expectations (Valtanen, 2019; Sidis et al., 2020) recognizing the importance of embodied presence, authenticity, shared understanding, shared responsibility, trust, and supervision in co-therapy practice. A unique contribution of the present research consists in highlighting how therapists’ individual presence, co-therapy processes and the shared space created dynamically interact inside and outside network meetings. Participants recognized an individual change as a result of a shared situation, making co-therapy a highly dynamic process and a transformational experience.

**Figure 1:**
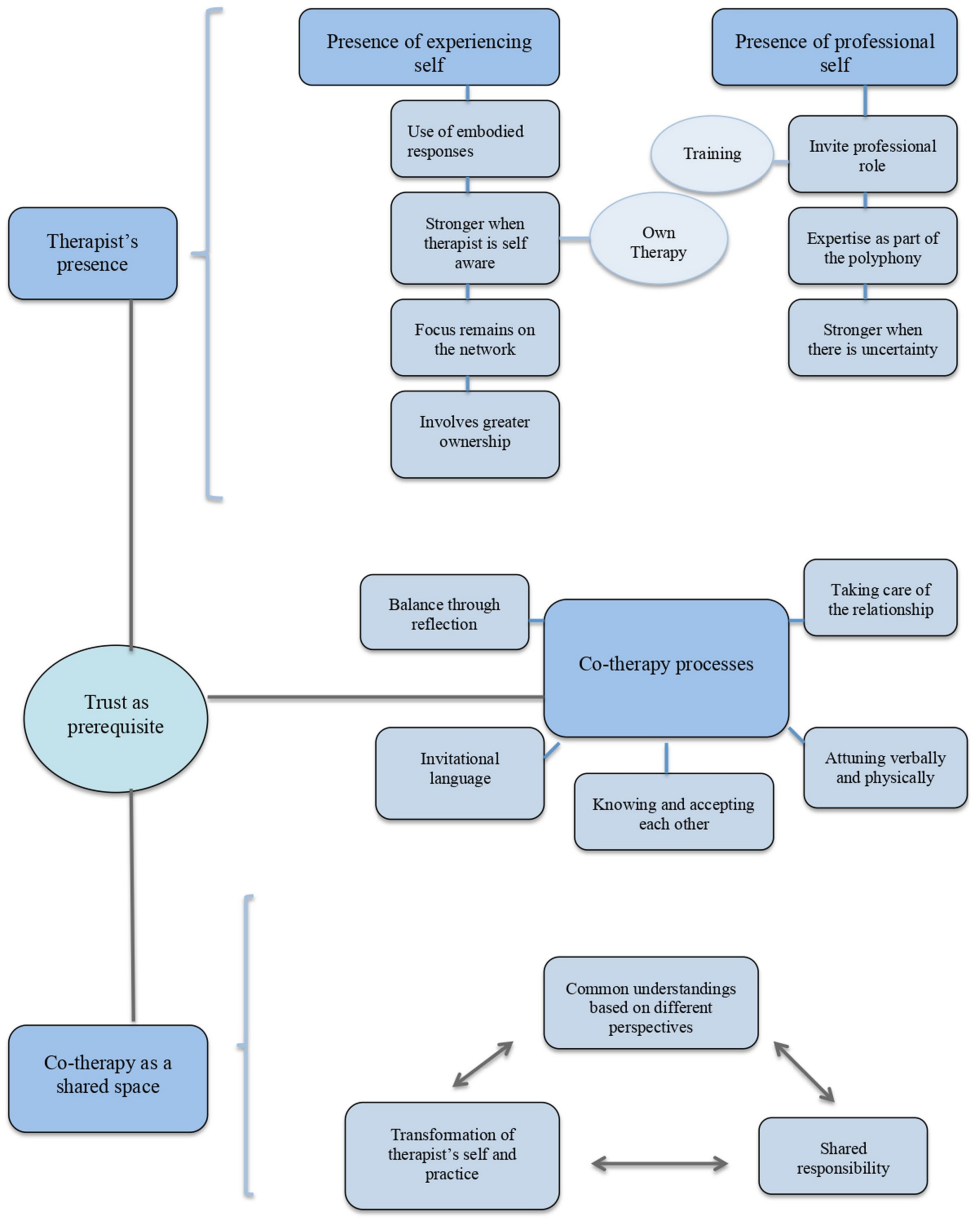
Dynamic relationship between therapist’s presence, co-therapy processes, co-therapy shared space and trust as a prerequisite in co-therapy practice.

Using thematic analysis participants’ experiences were captured in three main themes. Therapists are *present in a meeting with their experiencing and professional self* (Theme 1). Specific *co-therapy processes* allow co-therapists to tune in to each other verbally and physically (Theme 2). Through those processes a *shared space* is constructed that promotes new common understandings, shared responsibility and ultimately a transformation of each therapist’s self and practice (Theme 3). As illustrated in Figure 1, the quality of trust is woven throughout those themes, making trust a prerequisite for therapist’s individual presence, co-therapy processes and the shared space to unfold.

Research on therapist’s inner conversations during family therapy sessions with one therapist reveals the importance of therapists being attentive to their professional and experiencing self, as a way to respond to the families and create space for the not yet said (Rober, 2005a). Participants recognized that their experiencing self is present through their embodied responses and is stronger when they are self-aware. In line with previous research, self-growth practices, including personal therapy, family of origin, meditation etc., are critical for therapists to be aware of their own vulnerabilities and blind spots (Lum, 2002; Clark, 2009; Rowan and Jacobs, 2011; Ong and Buus, 2021). This helps them to keep the focus on the network and to make decisions regarding what voices of their experiencing self can become public or are worth exploring in different contexts. Weingarten (2010) suggested that this ability of therapists to be self-aware is accompanied by a sense of empowerment. In line with this, a participant in the present study said that bringing in the meeting one’s experiencing self involves greater ownership of their feelings and emotions. Sharing one’s emotions is not typical in traditional mental health trainings (Rowan and Jacobs, 2011), while there is an ongoing discussion about the opportunities and threats that come with self-disclosure and transparency in the field of family therapy (Roberts, 2005). Being more self-aware, through various practices, therapists develop a sense of owning their emotions and greater confidence, in that their remarks do not always have to be “right” but can come from the heart (Seikkula and Trimble, 2005).

The professional self, in terms of one’s professional expertise, is often the reason why specific practitioners are invited in meetings with networks. Still, all participants acknowledged that their professional self becomes another voice in the polyphony. This might explain why there were no differences in participants’ experiences of co-therapy despite their different original professional training. Similar to Hornova’s (2020) findings that co-therapists’ practices change in times of pressure and crisis, participants recognized that the voice of their professional self tends to be more dominant in times of uncertainty. Instead of viewing this uncertainty as a shared, overwhelming, experience of co-therapists, it seems that therapists opt for giving voice to each therapist’s concerns in turn. Breaking down therapists’ concerns is likely to make it easier to work through them and promote more opportunities for dialogue with the network. Specific co-therapy processes might help therapists regain trust in the therapeutic dialogue.

Participants repeatedly argued that what shapes their collaboration with their co-therapist is the personal ways of being with them, rather than their co-therapist’s professional background. Several co-therapy processes were mentioned. Co-therapists tend to balance each other through reflecting openly on their ideas and concerns. This is in line with literature suggesting that allowing time for reflection helps therapists not getting stuck in one position (Andersen, 1991, 1997; Borcsa and Janusz, 2021). Co-therapists can invite their co-therapists to unfold their thinking and share the reasons behind asking particular questions, addressing their professional self and the ways their stories may resonate to the networks’ narrative or addressing their experiencing self. It seems that using a language that focuses more on emotions and personal resonances rather than a language that tends to be dissociative and descriptive can further promote self-reflexivity and body-awareness for the therapist and the network (Hornova, 2020).

Knowing one’s co-therapists was acknowledged for most participants as key to being present in a meeting and attuning to each other in their embodied presence. This is in line with the limited research on co-therapists’ views of team meetings (Borchers et al., 2013; Holmesland et al., 2014; Hornova, 2020). Attention needs to be drawn, however, to the responsibilities that come with this familiarity (Borchers et al., 2013), as therapists might try to protect their co-therapist and/or avoid specific themes in a meeting that may be sensitive for their co-therapists, at the expense of the families’ exploration of alternative narratives. Future research needs to study further the challenges that come with therapists’ collaboration and familiarity.

Creating spaces outside the meeting to explore how it is for co-therapists to work together is key, as those spaces allow co-therapists to take care of their relationship, through exploring ways of being together. Supervision and the need for training have been widely recognized in the co-therapy practice in Marriage and Family Therapy research (Hendrix et al., 2001) and in Open Dialogue research (Hornova, 2020). Participants in the present study defined supervision as a space to explore ways of being with their co-therapist and develop opportunities to hold the space for each other, rather than as a way to discuss about the family and/or develop alternative hypotheses (Hendrix et al., 2001). Having such spaces to reflect on what co-therapists draw from the conversation with the network and how they want to address their co-therapist and the network members is critical for the creation of a safe therapeutic space and dialogue. Administrative and organizational structures need to protect co-therapists and provide the spaces for such dialogical and supervision practices, so that these become a learning experience for both the individual therapists and the co-therapy partners. This can be provided by service administration in the public sector but can be more demanding and costly in the private sector. For therapists working in the private sector greater initiative and commitment is required in offering themselves the supervision and reflective space to take care of the co-therapy relationship.

Through these co-therapy processes of balancing through reflections, taking care of the relationship, attuning verbally and physically, knowing and accepting each other and using invitational language co-therapists create a shared space that involves common understandings based on different perspectives. These common understandings contribute to the sense of shared responsibility between co-therapists and link back to two of the main principles of Open Dialogue meetings, namely allowing for responsibility and psychological continuity. Although sharing responsibility allows more flexibility in a meeting to explore feelings of curiosity and tolerate uncertainty in times of crisis, it may demand a role-expansion for some professionals (Holmesland et al., 2014; Hornova, 2020). Like all experiences involving change, one’s emotions are mixed, involving, among others, a sense of curiosity for the newness to come and a feeling of loss for what one leaves behind. Following participants’ testimonies, we propose that co-therapy can act both as a stimulus for such a transformational change and as a secure base to explore the multiple, often conflicting, feelings that accompany it.

Responsibility becomes a relational quality that is reflected and reflects in turn in the ways co-therapists are with a network (McNamee and Gergen, 1998). Creating a common understanding is very much based on co-therapists helping and inviting each other to openly share their thoughts and experiences, rather than competing with one another. Most importantly, it can be the case that if a therapist has not followed their co-therapist’s remarks, neither would the network members. Allowing the space to understand each other promotes the feeling of safety that therapists and network members are all in the therapeutic process together, having a shared language (Valtanen, 2019), which in turn contributes to the relationship between therapists and network members (Friedlander et al., 2006, 2011) and ultimately good therapeutic outcomes (Fife et al., 2014; Davis and Hsieh, 2019). This is in line with participants’ recognition that having common understandings with their co-therapist is the most important predictive factor for good outcomes in sessions.

In comparing poor and good outcomes of Open Dialogue, good outcomes have been associated with increased dialogical responses, compared to monological ones, in network meetings (Seikkula, 2002). As a therapist, the ability to promote dialogue depends not only on the training but most importantly on one’s dialogical personality (Brown, 2012; Reed, 2013; Brown et al., 2015). Cultivating a dialogical personality cannot solely rely on skills and techniques, but rather requires time and self-exploration to be accomplished; in this sense therapist’s dialogicity cannot be taught but can be learnt. Considering that Open Dialogue meetings are co-facilitated, this dialogical personality must be perceived in the context of the relationship with one’s co-therapist, rather than as an isolated personal characteristic. There has been some discussion around this transformation in dialogical literature, mainly concerning the ways practitioners perceive their professional identity and their expertise (Von Peter, 2021) and being authentic in voicing their feelings and emotions (Seikkula et al., 2012; Holmesland et al., 2014; Hornova, 2020). Drawing on all themes that emerged in the present study, participants highlighted that when working with a co-therapist the therapist’s inner conversation has an additional level concerning how their co-therapist responds to what is happening and how to use the space with the co-therapist to reflect on their own experiences. In this way co-therapy promotes a more dialogical personality and allows the therapist’s own transformation. This transformation enriches the positions of each therapist’s self in turn and allows for common understandings and sharing of responsibility.

### Limitations and recommendations

Apart from some research regarding co-therapists’ experiences of dialogical co-therapy (Hornova, 2020) and some empirical evidence on psychiatrists’ experience in teams with co-workers (Borchers et al., 2013), to our knowledge this is the first study to explore the professional self and experiencing self of therapists in a multi-dialogue context and their influences on co-therapy. Rober (2005a, 2017) studied therapist’s experiencing self and professional self using interpersonal process recall interviews, that are closely examining the therapist’s inner conversation retrospectively, 24 to 48 h after a meeting. The present study operationalized the professional and experiencing self differently, by following therapists’ own stories, experiences and meaning making of their professional role and lived experience in a network meeting. This might produce some discrepancy in the conceptualization of the different aspects of the self. It would be worth exploring the interplay and inner dialogue of co-therapists after an Open Dialogue meeting using interpersonal process recall interviews or semi-structured interviews with the co-therapist partners and explore if similar or different themes emerge.

Another limitation of the present study concerned the unbalanced number of participants’ professional backgrounds. There was an over-representation of psychologists in the research, only two psychiatrists, one psychiatric nurse and just one peer worker. Concerning the underrepresentation of peers in particular, there is a growing literature on the participation of peer workers in Open Dialogue meetings (Nelson et al., 2022; Osborne, 2022; Razzaque et al., 2022). Peer workers sharing aspects of their lived experience and voicing more of their experiencing self might draw different attention to the influence of professional and experiencing selves of co-therapists. This would be worth exploring further.

Although, participants recognized the value of allowing time to reflect on their relationship with their co-therapists, it would be of great interest if future research studied together co-therapists’ experience of their relationship in joint interviews or in a focus group. Reflecting together on their relationship was something suggested by some of the participants. A further insight in the dynamics and transformational value of co-therapy would come from networks’ perspectives on their co-therapists’ relationship and presence, through the use of questionnaires or an open conversation on “how we experience our co-therapists working together.”

### Conclusion

Co-therapy contributes significantly to the development of therapists’ professional self and experiencing self. Through various co-therapy processes, therapists support each other to attend to, unfold and share different voices of their inner dialogue with the network. It is easy to get stuck in one position, inspired by learnings of the professional self or by memories of the experiencing self. Through co-therapists’ invitation of one’s different voices therapists can make these voices less demanding, allow to move around different ideas and develop polyphony. This transformation of co-therapists in repositioning themselves as polyphonic individuals is reflected in the dialogical presence with each other that is not predetermined by their professional roles. Co-therapy can be a shared space for co-therapists to explore their differences and disagreements together, until something new emerges. This is how dialogue becomes healing.

## Data availability statement

The original contributions presented in the study are included in the article/supplementary material, further inquiries can be directed to the corresponding author.

## Ethics statement

The study was reviewed and approved by Open Dialogue UK. The participants provided their written informed consent to participate in the study.

## Author contributions

CL: conceptualization and original draft preparation. CL and DC: literature review. CL, EG, and DC: methodology, formal analysis, writing-review and editing. All authors have read and agreed to the published version of the manuscript.

## Conflict of interest

The authors declare that the research was conducted in the absence of any commercial or financial relationships that could be construed as a potential conflict of interest.

## Publisher’s note

All claims expressed in this article are solely those of the authors and do not necessarily represent those of their affiliated organizations, or those of the publisher, the editors and the reviewers. Any product that may be evaluated in this article, or claim that may be made by its manufacturer, is not guaranteed or endorsed by the publisher.
